# The Weight of Words: An analysis of Autobiographical Narratives and Psychopathological Measures in Anorexia Nervosa

**DOI:** 10.1007/s10936-025-10150-8

**Published:** 2025-05-08

**Authors:** Mariani Rachele, Massari Maria Giovanna, Renzi Alessia, Marini Isabella, Di Trani Michela, Pasquini Massimo

**Affiliations:** 1 Department of Dynamic and Clinical Psychology and Health Studies, Sapienza University, Rome, Italy; 2 Department of Human Sciences and Promotion of Quality of Life, San Raffaele Telematic University, Rome, Italy; 3 Dipartimento di Neuroscienze e Salute Mentale, AOU Policlinico Umberto I, Sapienza University, Rome, Italy; 4 Department of Human Neurosciences, Sapienza University , Rome, Italy

**Keywords:** Anorexia Nervosa, Linguistic Analysis, Autobiographical Narratives, Personality structures – psychopathology – mind/Body Disconnection

## Abstract

This study aims to explore the relationship between linguistic features of the Referential Process (RP) applied to autobiographical narratives, personality dimensions, and affect regulation capabilities in a group of women diagnosed with restrictive anorexia nervosa (AN). The study included 40 female participants hospitalized with AN during an acute phase, with a mean age of 19.50 (SD = 3.8). Participants completed several assessments, including the Minnesota Multiphasic Personality Inventory 2 (MMPI-2), the Eating Disorder Inventory (EDI-3), the 20-item Toronto Alexithymia Scale (TAS-20), the Emotion Regulation Questionnaire (ERQ), and the Relationship Anecdotes Paradigm Interview (RAP). The RAP interviews were audio-recorded and transcribed for the application of RP Linguistic Measures. The results of the correlation analysis revealed several significant associations among linguistic measures, EDI-3 scale scores, affect regulation measures, and personality dimensions. The linguistic measures indicating higher rationality, abstraction, and cognitive word usage, were associated with higher psychopathological severity in AN. Alexithymia showed significant correlations with the Affect words, supporting the perspective of MCT concerning dissociation of emotional schemas. These findings confirm the relationship between linguistic measures and the severity of the disease. Therefore, autobiographical narratives can be considered not only as diagnostic indicators, but also as variables to verify the efficacy of treatments in patients with AN.

## Introduction

Anorexia nervosa (AN) is an eating disorder characterized by self-induced restriction of caloric intake and significant body weight loss related to the fear of gaining weight and a distorted perception of one’s body image. Two subtypes can be distinguished: one characterized by the observance of a highly restrictive diet, sometimes resorting to fasting, and sometimes accompanied by continuous and exhausting physical activity (Type I), and another type characterized by binge eating followed by elimination behavior (Type II). The level of severity is assessed by the Body Mass Index (BMI), which, for diagnosis, must be significantly lower than the normal minimum (i.e., BMI < 18.5) (American Psychiatric Association & Association, 2013).

Within the spectrum of eating disorders, AN emerges as the most severe, characterized by a protracted course, and exhibits the highest mortality rate among all psychiatric illnesses (Resmark et al., 2019). It is crucial to emphasize that in Type I, the restriction of food intake often begins innocuously; however, over time, it evolves into a self-sufficient behavioral pattern (Walsh, 2013). This restrictive behavior is perceived as desirable and indicative of self-discipline, therefore experienced as ego-syntonic (Reas & Rø, 2018). Currently, the development of this disorder is attributed to a confluence of factors, encompassing biological, familial, and psychosocial elements. In this light, research and theory have suggested that significant variations in the onset, clinical course, symptom profile, and maintenance of eating disorders are connected with personality traits. Indeed, eating disorders are often associated with high perfectionism, neuroticism, and avoidance motivation, as well as lower extraversion and self-direction compared to control groups (Farstad et al., 2016).

In the Psychodynamic Diagnostic Manual- Second Edition (PDM-2) (Lingiardi & McWilliams, 2017), personality is defined as a set of relatively stable patterns of thinking, feeling, behaving, and relating to others. The Personality Patterns and Disorders axis of PDM-2, which conceives of personality as a dimensional concept, not only identifies personality style or type but also assesses the level of organization, indicating the severity of altered personality functioning (ranging from healthy to neurotic, borderline, and psychotic levels). The term ‘disorder’ therefore refers to a level of severity and rigidity such that functioning is impaired and causes suffering or difficulty. The most frequently diagnosed personality disorders, in comorbidity with AN, are borderline and paranoid disorders. Specifically, regarding Type I AN, the most commonly diagnosed personality disorders are avoidant and obsessive-compulsive personality disorders (Farstad et al., 2016).

It is possible to trace a neurotic structure underlying the functioning of patients with eating disorders, as well as psychotic cores. Defense mechanisms can be conceptualized along a continuum, ranging from immature to mature, influencing our perception of reality and our approach to problem-solving, thereby playing a critical role in the development and maintenance of eating disorders (Gitzinger et al., 1993). Specific research on the defensive functioning of patients with AN remains limited, with findings that are often inconsistent (Conversano et al., 2023). However, literature maintains that individuals with AN tend to use immature defense mechanisms more frequently than healthy controls (Gothelf et al., 1995). Recently, Craba et al. (2023) found that patients with AN overutilize more neurotic and primitive defense mechanisms compared with nonclinical subjects, and this use is associated with greater severity of eating symptomatology. In this regard, individuals with AN often deny their thinness, leading to a significant distortion of body image. Moreover, they also frequently deny the severity of their illness and their need for treatment (Bruch, 1982). This may be related to the extensive use of defense mechanisms typical of psychotic functioning (such as splitting, denial, and projective identification). Some studies showed that passive aggression, isolation, and devaluation are more frequently used by anorectic than bulimic patients (Stein et al., 2003; Costanzo et al., 2022), and indicate a worse profile of functioning. Therefore, it may be possible to hypothesize that AN could potentially evolve into a psychotic decompensation (Franzoni, 2004).

One method for comprehending how individuals articulate their experiences involves analyzing their speech patterns and the language they employ to narrate the experiences they encounter. The nuances of language offer insights into the capacity to connect emotions with verbal expressions and cognitive processes (Bucci, 1997b). In accordance with Bucci’s multiple code theory (MCT), a psychodynamic framework enabling the consideration of phenomena in both their physical and mental dimensions, along with a linguistic analysis of spoken or written text, enables the evaluation of connections between various methods or levels of processing and elaborating information (Bucci, 2001, 2021). These methods encompass: (1) the sub-symbolic system, which engages in simultaneous analogical processing of diverse forms of information, crucial for recognizing non-verbal communication and organizing the affective core; (2) the non-verbal symbolic system, which operates with discrete images or representations that emerge from the continuous processing of the sub-symbolic system; and (3) the verbal symbolic system, wherein images and representations can be transcribed into verbal form.

Life experiences encompass both symbolic and sub-symbolic forms, with memories organized in ‘memory schemas,’ which begin to form in early life, before language acquisition, and continue to develop throughout life. The memory schemas are active processes that can alter the way new events are experienced, and that can be modified by new events. According to Bucci, emotional experiences are also consolidated into specific memory patterns connoted by emotional experiences and visceral body patterns called emotion schemas. Just as memory patterns signify autobiographical experience, emotion schemas also signify patterns of visceral constellations and mnemonic representations that are specific to each individual (Mariani & Hoffman, 2021). Therefore, seeking to narrate personal and relational episodes entails tapping into emotional patterns and endeavouring to articulate and characterize experiences that are inherently intricate, ongoing, and multi-dimensional in nature.

According to the recent evolution of MCT (Bucci, 2021), the ability to connect emotional experience to verbal form is referred to as the Referential Process (RP). RP, as a whole process, occurs in three specific phases: (a) Arousal is an emotional activation generally connected to nonverbal experience related to memory of an emotional schema; (b) the Symbolizing function represents the moments when a connection between non-verbal information about the emotional experience is linked to a specific autobiographical memory in a verbal form, such as a representation of a relational interaction, a dream, an interpersonal experience, or a phantasy in relation to self or others; (c) the Reflection/Reorganizing function concerns a language that allows one to find new meanings in emotional experience. Generally, it is an experience of insight that allows one to understand one’s own activation of an emotional schema. These three functions, occurring sequentially, constitute what is termed the RP. They integrate subsymbolic non-verbal information with images and representations that can be expressed in words (Bucci, 2021). During highly traumatic or difficult life events, as in the case of AN, the activation of emotion schemas related to such events are more likely to produce unregulated and hard-to-manage arousal (Bucci, 2007, 2021; Renzi et al., 2023).

Bucci (2021) suggested that dissociation of the RP may indicate disconnections of emotional schemas so that what is coded in the non-verbal system is disconnected from the verbal symbolic system. In fact, acute external traumatic events can happen at any point in life, and longer-term issues related to the caregiving situation can also lead to dissociation and distortion within the emotion schemas. When faced with unpleasant or dangerous stimuli, people may avoid or disassociate from them, reasoning that these behaviors arise from typical biological reactions to similar situations. These responses occur at different times throughout the threat’s occurrence and in reaction to the threat. Within schemas, dissociation can manifest as either a distorted image of the object experienced as split off from the subsymbolic components of the affective core, or as arousal of the subsymbolic components of the affective core of terror with associated fight, flight, or freeze responses, without recognition or acknowledgment of the object that is the source of the activation. Dissociations of schemas are a consequence of disconnection among systems. Whether or not a specific trauma is detected, we contend that dissociation processes of the subsymbolic components from the verbal symbolic system are often the root cause of emotional disorders.

Starting with the results of a 2009 study (Ben-Meir et al., 2009), which used non-computerized measures for the evaluation of the RP, we aimed to carry forward the exploratory investigation into the relationship between language and AN disorder using computerized language measures. We additionally aimed to describe, both qualitatively and quantitatively, the relationship between AN severity and narrative characteristics in relation to the activation of the relational emotional patterns of the interviewees. Previous research (Mariani et al., 2022) applying RP measures to AN narratives showed a dissociation between mind and body: patients with AN utilize their bodies as a means of expressing emotions that elude mental and verbal representation. In fact, results revealed three linguistic as being strongly correlated with restrictive anorexia nervosa: sensory-somatic words, negative affect words, and a high RA intensity index (MH-IWRAD), representing Referential Process disconnection (Mariani et al., 2022). Furthermore, in our study, AN patients showed significant indices of Alexithymia and Emotional Dysregulation compared to the control group. Based on this work, we would expect to find associations among measures of RP disconnection, AN symptoms, and emotion regulation.

In this new, more specifically clinical investigation, we set out to explore within the group of patients with restrictive AN, the symbolization and reflection/reorganization processes of their own relational experiences in relation to the severity of the disorder, personality characteristics, and alexithymic processes. Specifically, we sought to answer the following exploratory questions:

1. Are there linguistic features in the narratives of autobiographical memories that we can consider prototypical of Restrictive Anorexia? In other words, are there any indicators that are associated with the severity of the disorder?
2. Is there a direct relationship between linguistic indices and emotion regulation (defined as the ability to feel and communicate emotional experience such as alexithymia) strategies in the AN clinical group?
3. Finally, is there a relationship between linguistic measures and psychopathological indices related to aspects of personality in the clinical group?

## Methods

### Participants

Participants were recruited from individuals seeking admission or visiting the Department of Psychiatry and Eating Disorders of the Umberto I Polyclinic, within its Inpatient Unit, Day Hospital, and Outpatient Clinic, according to the following inclusion criteria: a diagnosis of AN according to the SCID-5-CV semi-structured interview (First, 2014) conducted by an expert clinician; being between 16 and 30 years of age; currently experiencing the acute phase of AN; presenting with a BMI ≥ 12.5 kg/m2; and a diagnosis of AN for at least the past 6 months. Exclusion criteria include suffering from AN for ≥ 10 years; a diagnosis of acute psychosis; an intellectual disability; cognitive impairment; problematic use of alcohol or psychoactive substances; and/or any other conditions that might impact the understanding of questionnaires or the ability to provide informed consent.

The research sample consisted of 40 participants with a mean age of 19.40 (SD = 3.8) and a mean BMI of 16.11 (SD = 3.27). The educational background of participants was varied: 50% completed middle school, 37.5% completed high school, and the remaining 12.5% had attained an advanced degree. Of these participants, 90% were students, 5% were employed, and 5% were unemployed. 70% reported having undergone previous psychotherapy, while 30% reported no prior experience. Additionally, 40% of the participants disclosed a history of previous hospitalizations, while the remaining 60% reported no prior hospitalizations.

### Procedures

Data collection for this study took place from February 2021 to March 2023. Ethical approval for the study was granted by the Ethics Committee of the Department of Dynamic and the Clinical Psychology and Health Studies Department at Sapienza University of Rome. The research was conducted in compliance with the code of ethics of the World Medical Association (Declaration of Helsinki) for experiments involving human participants. The psychologist and the psychiatrist responsible for the protocol implementation screened the women for eligibility, and the psychologist obtained consent from the eligible women to participate in this study. Each participant with AN signed an informed consent form before completing the measures and the semi-structured interview. Self-report tests were administered using a specific Google form sent by email to participants who gave their consent. Subsequently, an interview was conducted online through the Meet software. The Relational Anecdotal Protocol interview method was administered by a qualified and experienced psychologist. The audio-recorded interviews were transcribed following specific procedures for the Italian version of the Discourse Attributes Analysis Program (DAAP) (Maskit & Murphy, 2011) for the computerized application of the RP linguistic measures.

### Measures

#### Socio-Demographic Questionnaire

This questionnaire collects information regarding age, level of education, relationship status, current occupation, and whether the individual is currently in psychotherapy.

#### The Minnesota Multiphasic Personality Inventory 2 (MMPI-2) (Butcher et al., 1989)

This is a standardized psychometric test used to study psychopathology and personality characteristics in adults. It consists of 567 dichotomously answered items (responded to as ‘true’ or ‘false’). The MMPI-2 is composed of three validity scales (the L scale, detecting attempts to provide false responses; the K scale, detecting defense and avoidance; and the F scale, indicating the frequency of atypical responses) and ten clinical scales (1-Hs hypochondria, 2-D depression, 3-Hy hysteria, 4-Pd psychopathic deviation, 5-Mf masculinity/femininity, 6-Pa paranoia, 7-Pt psychasthenia, 8-Sc schizophrenia, 9-Ma hypomania, and 10-Si social introversion). MMPI-2 is one of the most widely used tests for assessing major structural personality traits and major emotional disorders, both in psychological and psychiatric settings (Butcher & Williams, 2009). The Italian translation of the MMPI-2 has resulted in an instrument with very strong psychometric properties (Sirigatti & Stefanile, 2011).

#### The Eating Disorder Inventory (EDI-3) (Garner, 2004; Giannini et al., 2008)

This is a self-report questionnaire comprising 91 items on a 6-point Likert scale. It yields 12 main scales: three eating disorder-specific scales that provide information about attitudes and behaviors towards eating, weight, and body shape; and nine scales offering relevant information about psychological characteristics often present in patients with eating disorders. This instrument has demonstrated excellent reliability (α = 0.90) and validity in both clinical and control samples (Garner et al., 1982). In the present study, only the three eating disorder-specific scales (Drive for Thinness (DT), Bulimia (B), and Body Dissatisfaction (BD), as well as Eating Disorder Risk (EDRC), and General Psychological Maladjustment composite indexes (GPMC) were considered and analysed.

#### The Toronto Alexithymia Scale (TAS-20) (Bagby et al., 1994; Bressi et al., 1996)

This scale is the most widely used self-report measure for evaluating alexithymic characteristics in both clinical and non-clinical populations. Factor analysis reveals three distinct factors closely aligned with the construct: Difficulty Identifying Feelings (DIF), Difficulty Describing Feelings (DDF), and Externally Orientated Thinking (EOT). The TAS provides a score for each factor, as well as a total score. In the current study, Cronbach’s alpha value was 0.81.

#### The Emotion Regulation Questionnaire (ERQ) (Balzarotti et al., 2010; Gross & John, 2003)

This questionnaire comprises 10 items and encompasses two scales representing distinct emotion regulation strategies:

1. Cognitive Reappraisal (6 items): This scale assesses cognitive restructuring, a deliberate mental process aimed at modifying interpretations associated with emotional stimuli. Higher scores on this scale indicate effective utilization of this strategy to mitigate the impact of stressors;
2. Emotional Suppression (4 items): This scale evaluates attempts to inhibit the outward expression of emotions through both verbal and nonverbal cues. Higher scores on this scale indicate greater levels of emotional distress. For the present study, Cronbach’s alpha was found to vary between 0.76 and 0.85.

#### The Relationship Anecdotal Paradigm (RAP) (Luborsky, 1998)

This measure involves interviewees responding to the prompt, “Please tell me about incidents or events involving an interaction between yourself and another person” (Luborsky, 1998, p. 110). They are instructed to specify when the interaction occurred, with whom it occurred, provide details about what the other person said or did, and describe the eventual outcome. Interviewees are encouraged to share between 6 and 10 relationship episodes, and they have the freedom to recount incidents involving any person. Evidence supporting the validity of the RAP was presented by Barber, Luborsky, Crits-Christoph, & Diguer (Barber et al., 1995).

#### Linguistic Measures of the Referential Process

Building upon the multiple code theory (Bucci, 1997a), computerized language measures were employed to analyze the transcripts of referential process interviews using the Italian Discourse Attributes Analysis Program (IDAAP) software (Bucci et al., 2012; Mariani et al., 2013). The following linguistic measures were applied (see Table 1):

- *Italian Weighted Referential Activity Dictionary IWRAD* (Mariani et al., 2013): This dictionary contains 9596 frequently used linguistic elements and words belonging to the generative function of language, such as pronouns, articles, and prepositions. IWRAD enables microanalytic tracking of the Symbolizing phase of RP, depicting unintended components of emotional expressiveness, and assessing language style rather than content. See (Bucci et al., 2012) for a more thorough explanation of the process used to create a weighted dictionary, such as IWRAD.
  There are two other specific indexes connected to the Symbolizing phase:
  1. *Italian Mean High Weighted Referential Activity Dictionary (MH-IWRAD)*: Derived from IWRAD and also known as the Referential Activity Intensity Index, this index quantifies high-intensity emotional engagement expressed in speech (Mariani et al., 2013). The calculation involves counting IWRAD scores that exceed the neutral value, serving as a peak measure within the IWRAD scale.
  2. *The High Proportion IWRAD (HPIWRAD)*: This index represents the proportion of text in which the referential activity exceeds the neutral level.

- *The Italian Weighted Reflection and Reorganization List (IWRRL)*: The IWRRL evaluates a speaker’s ability to recognize and comprehend the experiential significance of events, focusing on emotional meaning rather than abstract reflection. It uses weighted Italian words associated with reorganization and reflection. Elevated scores on the IWRRL indicate heightened levels of such processes. As with IWRAD, two derivative indexes emerge for IWRRL:
  1. *The Mean High-Italian Weighted Reflection and Reorganization List (MH-IWRRL)*: is calculated by averaging the scores of these selected words;
  2. *The High Proportion of Italian Weighted Reflection and Reorganization List (HP-IWRRL)*: calculates the proportion of words where the IWRRL score exceeds the neutral value.

- *The Italian DisFluency Dictionary (IDF)*: The IDF is a concise list of 11 words used by individuals when facing communication challenges, including repeated, unfinished, and filled pauses (Bonfanti et al., 2008). The proportion of IDF terms in speech is reflected in the corresponding indicator score, with higher scores indicating activation of emotional schemas during periods of arousal.
- *The Italian Reflection Dictionary (IREF) (*Mariani et al., 2013): The IREF is a dictionary focused on cognitive processes and thought communication. It contains basic terms and phrases related to logical, cognitive, or impaired functions, which are characteristic of the reorganization phase.
- *The Italian Sensory Somatic Dictionary (ISenS)*: The ISenS is a list of vocabulary terms related to the body, physiological functions, sensory processes, and symptom descriptions (Bucci, 2021). The quantity of ISenS terms in a speech sample measures the arousal of physical, sub-symbolic components within emotion schemas.
- *The Italian Sum Affect Dictionary (ISAff)* (Mariani et al., 2013): The ISAff contains Italian words that directly express emotions and feelings. It includes emotion labels and functions associated with affective arousal. ISAff consists of three sub-dictionaries focused on affective domains: positive affect (IPAff), negative affect (INAff), and neutral affect without specific valence (IZAff).
- *The IREF_IWRAD Covariation*: This metric serves as the most direct indicator of the referential process’ functionality, measuring the quality of emotional processing undertaken by the subject. A negative covariation indicates the sequential, rather than simultaneous, presence of significant IWRAD and IREF values. During immersive narrative phases, IWRAD scores are high, while IREF are low; in the subsequent phase, when the patient takes a step back and reflects on the emotional significance of what has emerged, IREF are high, while IWRAD scores are low. The more negative the covariation, the greater the separation between these phases, indicating more effective processing.
  For an example of the words included in the dictionaries used, see Table 1.

**Table 1:** Examples of words belonging to referential process linguistic measures in the Italian Language

IWRAD	Italian Weighted Referential Activity Dictionary	IWRAD high weight: odore (smell = 0.981); maledetto (damned = 0.890); urla (scream = 0.835); stupore (astonishment = 0.786); baciare (to kiss = 0.747); IWRAD medium weight: il (the = 0.058); capisci (you understand = 0.0314); io (I = 0.018); lei (she = 0.008), ciò (that = -0.011); tu (you = -0.042) IWRAD low weight: superficiali (superficial = -0.870); parlavamo (we talked = -0.870); ansiosa (anxious =- 0.867); capiscono (they understand = -0.654); carini (nice = -0.519)
MH IWRAD	Italian Mean High Weighted Referential Activity Dictionary	It is called Referential Activity Intensity index and is calculated as the absolute score of the variable, i.c. its positive peak
HP IWRAD	High Proportion Weighted Referential Activity Dictionary	It is defined as the proportion of text that exceeds the 0.5 Referential Activity score, is calculated as a percentage
IREF	Italian Reflection Dictionary	Attenzione (attention), capire (to understand), decisione (decision), dubbio (doubt), meditare (to meditate), ragione (reason), razionalità (rationality), ricordo (memory).
IDF	Italian DisFluency Dictionary	Quindi (so), cioè (that is), comunque (however), allora (then), insomma (well), niente (nothing), magari (maybe), vabbè (don’t care), boh (don’t know), and ‘ehmm’ and ‘mm’ representing filled pauses with slightly different significance.
ISenS	Italian Sensory Somatic Dictionary	Ammalato (sick), digerire (digest), disorientamento (disorientation), dolore (pain), impotente (impotent), innervosito (unnerved), pesare (weigh), ridere (laugh), sintomi (symptoms), vomitare (throw up)
INAff	Italian Negative Affect Dictionary	Abbandonato (abandoned), depresso (depressed), impaurito (frightened), invidioso (envious), malinconia (gloom), odio (hate), sofferenza(suffering).
IPAff	Italian Positive Affect Dictionary	Abbracci (hugs), affidabile (reliable), baciare (to kiss), felice (happy), innamorato (in love), speranza (hope).
IZAff	Italian Neutral Affect Dictionary	Attesa (expectation), bisogno (need), coinvolto (involved), eccitato (excited) intensità (intensity), motivazione (motivation), reagire (react), sensazione (feeling).
ISAff	Italian Sum Affect Dictionary	Sum of all Positive, Negative, Neutral Affect Dictionaries as global affect list
IWRRL	Italian Weighted Reflection and Reorganization List	IWRRL high: schema (pattern), accorgersi (realizing), bisogni (needs), parlavo (I talked), adattarmi (adapting), pensare (to think), sapere (to know). IWRRL low: separazione (separation), chiamare (to call), scelta (choice), tale (such), finale (final), mancato (missed), intanto (while)
MH IWRRL	Italian Mean High Reflection Reorganization List	It is called Reflection-Reorganization Intensity index and is calculated as the absolute score of the variable, i.c. its positive peak
HP IWRRL	High Proportion IWRRL	It is defined as the proportion of text that exceeds the 0.5 Referential Activity score, is calculated as a percentage
IREF_IWRAD	Reflection and Referential Activity Covariation	It is defined as the covariation of two linguistic measures, reflexive-abstract words and Referential Activity, the more negative the inidice and the more distinguishable the symbolization and reflexive-reorganization phases.

### Data Analysis

The statistical analyses for the present study were completed using the Statistical Package for Social Science − 24 (SPSS version 24, Armonk, NY). Continuous variables are described as means and standard deviations, while discrete variables are reported as percentages and frequencies. Pearson’s correlation analysis was conducted to evaluate the associations between RP linguistic measures and eating disorder severity, affect regulation capabilities, and personality dimensions, respectively. A *p*-value of less than 0.05 was considered significant.

## Results

The tests scores of patients with AN are shown in Table 2. Regarding alexithymia scores, 60% of participants reported a TAS-20 total score above the clinical cut-off of 61, with a mean score of 61.23 (SD = 12.9). The EDI-3 severity index indicates that the patient group has severe AN, with symptoms resulting in severe impairment. Examination of general personality indices using MMPI-2 reveals that Depressive traits characterize the entire group of patients, followed by aspects of Hypochondria, Paranoia, and Schizophrenia (see Table 2).

**Table 2:** Psychological characteristics of the participants

	M	sd
**20-itemToronto Alexithymia Scale**		
Total score	61.23	12.91
Difficulty Identifying Feelings	23.64	6.69
Difficulty Describing Feelings	20.08	5.79
Externally Orientated Thinking	17.51	3.94
**Emotion regulation Questionnaire**		
Reappraisal	22.18	7.51
Suppression	18.51	5.34
**Eating Disorder Inventory-3**		
Thinness	82.19	20.57
Bulimia	49.14	34.21
Body Dissatisfaction	77.69	17.32
Eating Disorder Risk scale	77.89	16.63
General Psychological Maladjustment	81.31	18.75
**Minnesota Multiphasic Personality Inventory 2**		
“Lie” / Uncommon Virtues	49.88	11.56
Infrequency	62.84	11.26
Defensiveness	44.38	9.74
Hypochondria	68.56	11.59
Depression	75.47	12.36
Hysteria	64.28	11.56
Psychopathic deviation	64.50	10.42
Paranoia	66.81	12.82
Psychasthenia	67.66	10.48
Schizophrenia	67.47	10.63
Hypomania	56.16	10.40
Social introversion	61.03	11.46
Masculinity/femininity	40.91	7.69

In Table 3, the associations between RP linguistic measures and eating disorder severity, as evaluated by EDI-3 scales and BMI, are presented. No significant associations between RP measures and BMI emerged; however, several significant associations with EDI-3 dimensions were found (see Table 3).

**Table 3:** Associations between referential process linguistic measures, eating disorder Inventory-3 scales, and body mass index

	BMI	EDI3 DT	EDI3 B	EDI3 BD	EDI3 EDRC	EDI3 GPMC
Words	0.164	0.030	-0.046	0.290	0.102	0.098
IDF	0.044	0.029	-0.047	-0.090	-0.084	-0.011
INAff	0.077	0.206	0.017	0.143	0.152	0.267
IPAff	-0.034	-0.125	0.094	0.036	0.032	0.044
ISAff	0.012	0.002	0.115	0.176	0.130	0.293
IZAff	-0.074	-0.162	-0.001	0.037	-0.138	0.032
IREF	0.124	0.270	0.159	0.362^*^	0.276	0.367^*^
ISenS	-0.089	-0.051	− 0.368^*^	-0.058	-0.066	0.241
IWRAD	0.018	-0.067	0.141	-0.131	0.002	-0.002
IWRRL	0.012	0.035	-0.089	0.415^*^	0.144	0.266
MH IWRAD	0.040	-0.162	0.086	-0.238	-0.120	-0.140
HP IWRAD	0.024	0.008	0.123	-0.103	0.043	0.088
MH-IWRRL	0.008	0.048	-0.103	0.404^*^	0.149	0.270
HP IWRRL	0.059	-0.156	0.174	0.351^*^	0.031	0.115
IREF_IWRAD	0.146	0.170	0.004	0.266	0.177	0.083

*Legend*. BMI = Body Mass Index; EDI-3 = Eating Disorder Inventory; DT = Thinness; B = Bulimia; BD = Body Dissatisfaction; EDRC = Eating Disorder Risk scale; GPMC = General Psychological Maladjustment; IDF = Italian Disfluency Dictionary; INAff, IPAff, ISAff, IZAff = Italian Dictionary of, respectively, Negative, Positive, Sum, neutral (Z) Affects; IREF = Italian Reflection Dictionary; ISenS = Italian Sensory-Somatic; Dictionary; IWRAD = Italian Weighted Referential Activity Dictionary; IWRRL = Italian Weighted Reflection and Reorganization List; MHIWRAD = Italian Referential Activity Intensity Index; HPIWRAD = High Proportion IWRAD; MH-IWRRL = Mean High–Italian Weighted Reflection and Reorganization List; HPIWRRL = High Proportion IWRRL; IREF_IWRAD = Covariation Italian Reflection Dictionary Italian Weighted Referential Activity Dictionary

^*p<.05; **p<.01^

In Table 4, the correlations between RP linguistic measures and alexithymia and emotion regulation capabilities are reported. Significant associations with both affect regulation capabilities measures emerged (see Table 4).

**Table 4:** Correlations between referential process linguistic measures, alexithymia, and emotion regulation

	TAS-20 TOT	TAS-20 DIF	TAS-20 DDF	TAS-20 EOT	ERQ Cognitive Reappraisal	ERQ Emotional Suppression
Words	-0.100	0.172	-0.309	-0.191	-0.126	-0.174
IDF	0.050	0.278	-0.170	-0.076	-0.223	-0.099
INAff	-0.055	-0.068	-0.131	0.129	-0.241	0.167
IPAff	0.347*	0.167	0.391*	0.303	-0.148	0.037
ISAff	0.335*	0.170	0.273	0.433**	-0.394*	0.215
IZAff	0.061	0.193	-0.085	-0.010	-0.077	0.095
IREF	-0.032	0.113	-0.162	-0.070	-0.547**	-0.254
ISenS	0.288	0.359*	0.154	0.108	-0.298	0.299
IWRAD	-0.108	-0.274	0.090	-0.011	0.051	-0.066
IWRRL	0.129	0.180	0.035	0.067	-0.272	0.088
MH IWRAD	-0.090	-0.140	0.032	-0.104	0.108	0.005
HP IWRAD	-0.034	-0.251	0.157	0.100	0.029	-0.011
MH IWRRL	0.137	0.189	0.037	0.074	-0.260	0.105
HP IWRRL	-0.007	-0.002	0.032	-0.068	-0.268	-0.135
IREF_IWRAD	0.187	0.076	0.343*	-0.007	0.093	0.042

*Legend*. TAS-20 = 20-itemToronto Alexithymia Scale; DIF = Difficulty Identifying Feelings; DDF = Difficulty Describing Feelings; EOT = Externally Orientated Thinking; ERQ = Emotion regulation Questionnaire; IDF = Italian Disfluency Dictionary; INAff, IPAff, ISAff, IZAff = Italian Dictionary of, respectively, Negative, Positive, Sum, neutral (Z) Affects; IREF = Italian Reflection Dictionary; ISenS = Italian Sensory-Somatic; Dictionary; IWRAD = Italian Weighted Referential Activity Dictionary; IWRRL = Italian Weighted Reflection and Reorganization List; MHIWRAD = Italian Referential Activity Intensity Index; HPIWRAD = High Proportion IWRAD; MH-IWRRL = Mean High–Italian Weighted Reflection and Reorganization List; HPIWRRL = High Proportoin IWRRL; IREF_IWRAD = Covariation Italian Reflection Dictionary Italian Weighted Referential Activity Dictionary

^*p<.05; **p<.01^

Regarding the associations between RP linguistic measures and MMPI-2 personality dimensions, several significant correlations emerged with both with the Lie (L) and Infrequency (F) validity scales, as well as with different clinical scales (see Table 5).

**Table 5:** Correlations between referential process linguistic measures and the scales of the Minnesota multiphasic personality Inventory-2 showing significant associations

	MMPI L	MMPI F	MMPI Pd	MMPI Pa	MMPI Pt	MMPI Sc	MMPI Ma	MMPI Mf
Words	0.025	0.182	0.067	0.174	0.006	0.163	0.040	0.078
IDF	-0.475**	0.015	0.009	0.232	0.177	0.037	-0.013	0.123
INAff	0.076	0.068	0.144	0.295	0.226	0.141	-0.375*	-0.280
IPAff	-0.273	0.276	0.082	0.020	0.175	0.283	0.342	0.035
ISAff	-0.273	0.453*	0.277	0.369*	0.409*	0.524**	0.134	-0.159
IZAff	-0.185	0.351	0.214	0.290	0.065	0.345	0.394*	0.211
IREF	− 0.375*	0.126	0.405*	0.229	0.282	0.331	-0.065	-0.132
ISenS	-0.146	0.180	0.043	0.237	0.124	0.229	-0.160	− 0.363*
IWRAD	0.308	-0.232	0.119	-0.239	-0.067	-0.137	-0.303	-0.206
IWRRL	-0.179	0.301	0.148	0.299	0.144	0.428*	0.119	0.140
MH IWRAD	-0.050	-0.196	0.000	-0.166	-0.013	-0.135	-0.069	-0.130
HP IWRAD	0.396*	-0.168	0.131	-0.182	-0.091	-0.138	-0.358	-0.232
MH IWRRL	-0.184	0.313	0.144	0.315	0.166	0.446*	0.115	0.139
HP IWRRL	-0.022	-0.015	0.119	-0.070	-0.251	-0.054	0.175	0.068
IREF_IWRAD	0.193	0.031	-0.172	0.032	0.061	0.164	-0.131	-0.064

*Legend*. IDF = Italian Disfluency Dictionary; INAff, IPAff, ISAff, IZAff = Italian Dictionary of, respectively, Negative, Positive, Sum, neutral (Z) Affects; IREF = Italian Reflection Dictionary; ISenS = Italian Sensory-Somatic; Dictionary; IWRAD = Italian Weighted Referential Activity Dictionary; IWRRL = Italian Weighted Reflection and Reorganization List; MHIWRAD = Italian Referential Activity Intensity Index; HPIWRAD = High Proportion IWRAD; MH-IWRRL = Mean High–Italian Weighted Reflection and Reorganization List; HPIWRRL = High Proportion IWRRL; IREF_IWRAD = Covariation Italian Reflection Dictionary Italian Weighted Referential Activity Dictionary; MMPI-2 = Minnesota Multiphasic Personality Inventory 2; L “Lie” / Uncommon Virtues; F Infrequency; Pd = psychopathic deviation; Pa = paranoia; Pt = psychasthenia; Sc = schizophrenia; Ma = hypomania; Mf = masculinity/femininity

^*p<.05; **p<.01^

## Discussion

In this study, we explored the linguistic aspects of the RP within a sample of patients with severe AN. Our first research goal was to explore the relationship between RP linguistic measures and the severity of AN measured by the EDI scale. The theoretical model of reference is MCT, which suggests that in severe disorders such as AN, when soliciting autobiographical life narratives, difficult connections between affective aspects and narrative style can be evidenced.

We hypothesized that the severity of AN would be reflected in the language indices. No linear relationship emerged between Body Mass Index and linguistic measures. However, correlations with psychopathological scales of EDI scales and linguistic measures of RP revealed some interesting findings. The two linguistic measures that showed greater relationships with the psychopathological severity indices of AN were the Reflection Dictionary (IREF) and the Weighted Reflection and Reorganization List (IWRRL) (see Table 3). Specifically, when examining the correlations between IREF and the EDI-3 Global scale, we found that the autobiographical narratives of the interviewed patients exhibited a close relationship between high rational, abstract, and intellectualized words and symptom severity. IREF has been shown to be a very effective index of psychopathology in a non-clinical population during the COVID-19 lockdown (Mariani, Monaco & Di Trani, 2021).

IWRRL displayed a positive relationship to Body Disaffection (EDI3 BD). These findings highlight the close relationship between abstraction processes and the emotional experience of patients with AN. It seems as if the process of devaluing the body turns out to be a signifier of the symptomatic process, i.e. attacking and denigrating one’s body performs a function of reorganizing experience as if to justify one’s symptom. Other research found similar results indicating that patients with high AN use intellectualization more frequently than normal adolescents and psychiatric patients. Individuals with AN further use cognitive processes more than other psychiatric patients in attempting to understand and explain reality (Gothelf et al., 1995).

The Bulimia Index (EDI3 B) showed a negative correlation to the Sensory-Somatic Dictionary. This result is unexpected; one might in fact think that the greater the impulse to eat, the greater the reference to the sensory-somatic experience. On the contrary, the negative correlation seems to emphasize verbalization instead of the acted-out symptom. That is, talking about one’s sensory-somatic experiences reduces food-seeking and promotes restrictive behavior in this population.

Our second research goal was to explore the relationship among RP, alexithymia, and emotion regulation strategies in patients with AN. In our clinical sample, it is evident that the AN population surveyed shows a sample mean higher than the cut-off, thus highlighting a high level of difficulty in perceiving and recognizing one’s own emotions (Table 1). The results showed significant correlations between Alexithymia and the Affect Dictionary. At first glance, this element seems to be contradictory, as it seems to challenge the idea that these patients are capable of expressing emotions. However, according to MCT, naming emotions differs from recognizing and perceiving them. In fact, emotionally engaging, symbolized experiences often lack explicit affective labels. Instead, they convey significant autobiographical narratives through which both the narrator and, more importantly, the listener, can share the same emotional experience (Di Trani et al., 2018; Mariani et al.,, 2020). We note that while the findings showed a positive correlation with the Affect dictionaries, there was no correlation with the IWRAD measures, which, according to MCT, are the indicators of communicating emotional experiences through narratives. The low level of IWRAD and higher use of Affect words would thus be consistent with the concept of Alexithymia. Furthermore, the negative correlation between the ERQ cognitive-reappraisal and the Sum of Affects (*r*=-.394; *p* =.019) indicates a dysfunctional element. Similarly, the strong negative correlations between I-REF and the ERQ cognitive-reappraisal (*r*=-.547; *p* =.001) emphasizes the dysfunctional regulatory function of abstraction and intellectualization in patients with AN. These individuals use abstraction and intellectualization as an attempt to signify and explain something that cannot be deeply activated within their narrative.

The Italian Sensory Somatic Dictionary showed a positive association with Difficulty in Identifying Feelings (*r* =.359; *p* =.034) and the covariation between the Reflection Dictionary and Weighted Referential Activity (IREF_IWRAD), which was positively correlated with Difficulty in Describing Feelings (*r* =.343; *p* =.044). This can be seen as another indicator of the failure of the RP in regulating and communicating emotion in individuals with AN. Similarly, the Italian Somatic Sensory Dictionary was positively correlated with Difficulty in Identifying Feelings (*r* =.359; *p* =.034) and IREF_IWRAD was positively correlated with Difficulty in Describing Feelings (*r* =.343; *p* =.044). The IREF_IWRAD index measures symbolic activation when negative. This positive correlation confirms the disconnection process in cases of severe AN. This relationship also shows that the symbolic processes in autobiographical interviews are not activated in a useful way. The use of abstract words, as well as the use of words naming affects, or words indicating bodily symptoms would seemingly take the place of real emotional activation.

Finally, our last research aim encompassed an exploratory investigation within this clinical group between linguistic measures and psychopathological traits. Many studies have been completed trying to identify if there are specific personality disorders associated with restrictive AN. It has often been researched whether AN is linked to histrionic or narcissistic traits. However, our investigation (Table 1) suggests the presence of multiple psychopathological traits, indicating that no single personality disorder distinctly differentiates the group of individuals with AN.

We then examined whether linguistic measures correlated with specific psychopathological traits (MMPI-2) and found an interesting relationship with a particular control scale of the MMPI-2 (see Table 5). Specifically, High Proportion IWRAD (HP IWRAD) showed a positive correlation with the MMPI-2 Lie Social Desirability Scale. This scale introduces an element of control of the test and its validity, influencing the portrayal of self-image. This finding suggests an effort to engage the listener during the interview. Conversely, the Disfluency measure, which escapes the control of linguistic eloquence, also negatively correlates with the MMPI-2 Lie scale.

According to these measures, greater fluency indicates a greater amount of lying and inauthenticity. These findings align with previous research indicating that individuals with AN struggle with issues of authenticity (Hope et al., 2011). This observation is particularly significant given the widespread reliance on self-report measures in clinical samples (see Table 6). The tendency to lie raises questions about the reliability of self-report measures in individuals who struggle to recognize aspects of their emotional experiences, which aligns with the findings of other studies (Mariani et al., 2020). Additionally, for a more adequate clinical assessment, it is crucial to consider the extent to which control over food does not also correspond with control over one’s emotions in this population. In other words, individuals in this population may control their food intake to compensate for not being able to control of the emotions they experience.

**Table 6:** Basic measures of RP for the RAP in patients with AN and in normative comparative populations in prior studies

	AN Patients (*n* = 40)		Control group AN Mariani et al. (2022) (*n* = 36; F = 36; mean age = 21; sd = 2.4)		Control group depression Mariani et al. (2020) (*n* = 20; F = 10; M = 10; mean age = 47.10; sd = 16.80)	
	M	SD	M	SD	M	SD
Words	1916.25	1020.427			4695.60	1298.49
IDF	0.092	0.036	0.092	0.023	0.053	0.026
INAff	0.015	0.007	0.011	0.004	0.010	0.003
IPAff	0.017	0.008	0.013	0.005	0.012	0.005
ISAff	0.036	0.007	0.029	0.007	0.026	0.008
IZAff	0.004	0.002	0.004	0.002	0.004	0.001
IREF	0.030	0.008	0.033	0.009	0.026	0.005
ISenS	0.044	0.011	0.035	0.007	0.036	0.004
IWRAD	0.504	0.003	0.501	0.003	0.504	0.004
IWRRL	0.543	0.004				
MH IWRAD	0.010	0.002	0.008	0.002	0.010	0.002

*Legend*. IDF = Italian Disfluency Dictionary; INAff, IPAff, ISAff, IZAff = Italian Dictionary of, respectively, Negative, Positive, Sum, neutral (Z) Affects; IREF = Italian Reflection Dictionary; ISenS = Italian Sensory-Somatic; Dictionary; IWRAD = Italian Weighted Referential Activity Dictionary; IWRRL = Italian Weighted Reflection and Reorganization List; MHIWRAD = Italian Referential Activity Intensity Index

These results are highly relevant because they demonstrate that freely spoken, open-ended narratives allow for a more authentic and effective means of assessment than self-report tests, particularly in clinical populations with severe conditions. In exploring the relationship between the MMPI-2 and linguistic patterns, we find further confirmation that a higher frequency of negative affective labels is indicative of more severe symptoms, particularly depression - a finding emphasized in a previous study (Mariani et al., 2020). Furthermore, this group of patients exhibits a pervasive depressive trait.

Finally, the indices of reflection, reorganization, and the use of abstract words in this specific sample highlight strong relationships with traits such as hypochondria and distortion of reality; elements which therefore suggest a thought disorder. This element underscores even more how pseudo-mentalization can develop as an attempt to compensate for the difficult contact with one’s emotions (Stavrou, 2021).

Returning to the title of our work, “The Weight of Words,” for patients with AN, this phrase takes on a particular ethereal characteristic and showcases that an absence or distortion of contact with one’s own weight highlights a delicate defensive process.

The present findings need to be interpreted in light of some limitations. First, the sample size is relatively small, partially due to the decision to focus on a clinical sample. Second, the study is limited by its gender homogeneity, as all participants were female. This is partially consistent with the clinical population, which is predominantly represented by women, with men representing a smaller proportion. However, future studies exploring these dimensions in male patients would be highly valuable. Another limitation is the absence of a comparison group, both of healthy controls and of individuals with other psychopathological conditions, which would help determine whether the present findings are specific to this condition or shared with other populations. A further limitation is related to the high number of correlations tested, so the results should primarily be considered for their descriptive value and overall consistency. Finally, the use of self-report measures may introduce bias in evaluating the constructs under investigation.

## Conclusion

This study focused on language style and referential process analysis in patients with restrictive AN. In particular, we highlighted how certain linguistic aspects are closely related to clinical symptom severity. Abstract words and attempts to reflect upon and organize experience are closely related to symptom severity. This element is noted in many studies of anorexia, where the intellectualized use of experience turns out to be a defensive way of coping with life experience. The strong difficulties of being in touch with emotional experience given the high levels of alexithymia and emotional dysregulation present in the sample turn out to be associated with a narrative style focused on the body, somatic perception, and symptom description.

Autobiographical narration of one’s own significant relationships in this group of patients brings out relevant difficulties in linking emotions to the narrative account by highlighting elements of disconnection of the patterns of emotion. The close relationship between alexithymia and difficulties in symbolizing emotional experience seem to highlight this very element. In addition, linguistic measures applied in a clinical setting provide elements that are spontaneous and unintended by the speaker; thus bringing out deeper elements of his or her emotional organization. The close relationship between Referential Activity and the lie scales of the MMPI emphasize to us the strong limitation of basing investigations on self-report instruments.

We can therefore argue that linguistic analyses can provide innovative tools for refining our clinical understanding of patients and enable access to symbolic and reflexive processes that help us understand key elements of emotional functioning.

This is particularly relevant in this context, as patients are undergoing a multidisciplinary intervention which includes psychotherapy. Therefore, for clinicians involved in treating this specific population, the present findings suggest the importance of considering how patients with AN narrate their experiences. This narrative style may serve as a means to evaluate the course of the disease, potentially indicating a trajectory toward recovery or, conversely, a relapse into greater severity.
